# Determination of the endothelin-1 recognition sites of endothelin receptor type A by the directed-degeneration method

**DOI:** 10.1038/s41598-017-08096-6

**Published:** 2017-08-08

**Authors:** Seong-Gu Han, Sanghwan Ko, Won-Kyu Lee, Sang Taek Jung, Yeon Gyu Yu

**Affiliations:** 1 Department of Chemistry, Kookmin University, 861-1 Jeongneung-dong, Seongbuk-gu, Seoul, 136-702 Republic of Korea; 2 New Drug Development Center, Osong Medical Innovation Foundation, Osong Sengmyung-Ro 123, Osong-eup, Heungdeok-gu, Cheongju-si, Chungbuk Republic of Korea

## Abstract

G-protein coupled receptors (GPCRs) play indispensable physiological roles in cell proliferation, differentiation, and migration; therefore, identifying the mechanisms by which ligands bind to GPCRs is crucial for developing GPCR-targeting pharmaceutics and for understanding critical biological functions. Although some structural information is available regarding the interactions between GPCRs and their small molecule ligands, knowledge of how GPCRs interact with their corresponding macromolecule ligands, such as peptides and proteins, remains elusive. In this study, we have developed a novel strategy to investigate the precise ligand recognition mechanisms involved in the interaction of endothelin receptor type A (ET_A_) with its ligand, endothelin-1 (ET-1); we call this method “directed degeneration” method. Through flow cytometric screening of a randomized ET_A_ library, statistical analysis of the identified sequences, and biochemical studies, the ligand interaction map was successfully obtained.

## Introduction

G-protein coupled receptors (GPCRs) are the largest membrane protein family; they regulate various crucial physiological processes, including cell migration, differentiation, and proliferation, by delivering extracellular signals into the interior of the cell by triggering the activation of G-protein heterotrimers^1–3^. The signaling cascade of a GPCR is initiated by binding to its ligand. Therefore, identifying the ligand recognition mechanisms of GPCRs is crucial for facilitating drug discovery processes as well as elucidating signal cascades. Understanding the thermodynamics of binding of a ligand to a target GPCR, for example, has guided the development of new drugs by rational approaches^4–12^. Furthermore, several reports have suggested that optimization of the ligand binding enthalpy may address ADME issues (adsorption, distribution, metabolism, and excretion of a drug)^10–13^. Dozens of GPCR complex structures with small molecule ligands have revealed how GPCRs recognize small molecule ligands^14^; these structures indicate that the binding pockets of small molecule ligands are located in the transmembrane regions (TMs) of GPCRs. However, the binding mechanism of native macromolecule agonists, such as endothelin-1 (ET-1) and Wnt to their corresponding GPCRs remains elusive.

Human endothelin receptor type A (ET_A_) is a Class A (rhodopsin-like) GPCR that is involved in vasoconstriction *via* a G_αq_ signaling cascade triggered by binding its native peptide agonist, ET-1^15^. Recently, the correlation between ET_A_ and the progression of various cancers by increasing metastatic potential and proliferation has been validated^3, 16–18^. Furthermore, the expression of endothelin receptors in cancer cells reduces patient survival rate by promoting cancer malignancy^19^. Due to this close relationship between ET_A_ and tumor malignancy, ET_A_ is an attractive cancer drug target. For example, bosentan, a drug currently available on the market that targets endothelin receptors for pulmonary arterial hypertension, has been re-developed for the treatment of melanoma^3^. Therefore, understanding the ligand recognition of ET_A_ is highly important to facilitate the development of drugs targeting ET_A_ as well as to understand the signal cascades of the receptor.

The ET-1 binding sites of ET_A_ have been studied using mammalian cells expressing chimeric ET_A_^20, 21^ as well as by molecular modeling^22–24^. However, the results are ambiguous, or the binding sites are too localized to explain the global binding mode of ET-1 against ET_A_. For example, Wallace and his colleagues showed that ET-1 binds to the extracellular domain of ET_A_ according to their molecular model^22^, whereas Rao *et al*. suggested that the transmembrane domain of ET_A_ is the ET-1 binding pocket^23^. Therefore, a precise and global ET-1 interaction map of ET_A_ is necessary to understand the ET-1 recognition mechanism.

**Figure 1:**
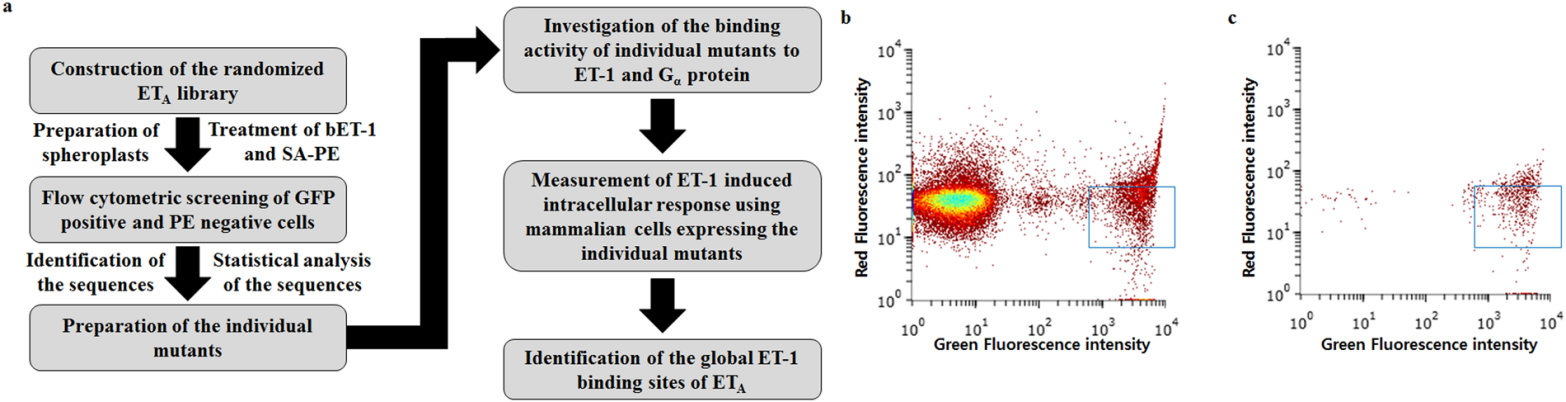
The directed degeneration method and flow cytometric screening of the ET_A_ library. (**a**) Flow chart of the directed degeneration method. After construction of the ET_A_ library by random mutagenesis, the spheroplasts expressing the library were treated with bET-1 and SA-PE, followed by isolation of the GFP-positive and PE-negative clones using FACS. After sequence analysis of the isolated clones, the P9-ET_A_s harboring these mutations were prepared, and their binding affinities to ET-1 were characterized. Also, the effects of the isolated mutations on ET-1-dependent signaling were confirmed in CHO-K1 cells expressing the ET_A_ mutants. Through biochemical and cell-based studies, the ET-1 interaction map of ET_A_ was successfully generated. (**b** and **c**) Histograms showing the first round (**b**) and the second round (**c**) of FACS screening of the ET_A_ library. The blue squares in the histograms indicate gating regions. In the first and the second rounds of sorting, approximately 10^6^ spheroplasts comprising 4% and 1.4% of the total population were collected.

Recently, we established an *E. coli* expression system of ET_A_ by N-terminus fusion of P9, which is an envelope protein of *Pseudomonas* phi6, and an amphipathic polymer that can stabilize membrane proteins in their functional conformation; these are called the P9 expression system and amphipathic poly-γ-glutamic acid (APG), respectively^25–28^. Although the ET_A_ had N-terminal P9-tag without glycosylation, the protein showed specific and selective binding activities with its ligand and G_α_ proteins indicating that this system should be appropriate for screening of ET_A_ variants for ET-1 binding. With these methods, we identified the precise ET-1 binding mechanism of ET_A_ with a novel biochemical strategy called the “directed degeneration” method as shown in Fig. 1a. First, we generated a randomized ET_A_ library by error-prone PCR. We then screened the degenerative clones using fluorescence activated cell sorting (FACS) to isolate the clones that had lower ET-1 binding activity compared to wild type ET_A_ and had no internal stop codon. Through statistical sequence analysis of the isolated clones followed by biochemical analysis of the purified ET_A_ mutants, the ET-1 interaction map of ET_A_ was successfully obtained. The effects of these mutations on intracellular signaling were finally confirmed by measuring ET-1 induced variations of intracellular Ca^2+^ levels using mammalian cells expressing the mutants. Here, we demonstrate that the extracellular N-terminus region and the entrances of the third and seventh transmembrane helix of ET_A_ are involved in the interaction with ET-1.

## Results

### Construction of a randomized ET_A_ library and isolation of ET_A_ variants exhibiting reduced binding to ET-1

Through randomization of ET_A_ and fusion of P9 and green fluorescence protein (GFP) at the N-terminus and C-terminus, respectively, an ET_A_ library composed of approximately 10^3^ diverse ET_A_ variants was successfully constructed. As shown in Supplementary Table S1, the average mutation number per clone was approximately 5.9. Considering the size of the ET_A_ (2 to 427) gene (1278 bp) and the average mutation number per clone, the error rate of the library was estimated to be 0.46%; this is in good agreement with the desired error rate of the error-prone PCR step (0.5%).

After cultivation of the library, the outer membrane and peptidoglycan layer of the cells, expressing the randomized ET_A_ fused with P9 and GFP at the N-terminus and C-terminus, respectively, were removed; the resulting spheroplasts were sequentially incubated with 0.5 μM C-terminally biotinylated ET-1 (bET-1) and 10 nM phycoerythrin (PE)-conjugated streptavidin (SA-PE). The spheroplasts that exhibited positive GFP fluorescence due to the absence of an internal stop codon and negative PE fluorescence due to their low ET-1 binding activity were selectively collected by two flow cytometry sorting processes (Fig. 1b and c). As shown in Fig. 1b, a substantial portion of the ET_A_ library showed very low GFP signals, suggesting that these clones may have internal stop codons resulting from random mutagenesis during the library construction. Through the first round of sorting, the cells that showed high GFP signals and low PE signals, representing approximately 4% of the total applied cells (Fig. 1b, boxed region), were isolated. The results of the subsequent sorting round showed that most of the clones with low or mid-range GFP signals and high PE signals were successfully eliminated (Fig. 1c). Through the second sorting, approximately 10^6^ GFP-positive and PE-negative cells, representing 35% of the first isolated cells and 1.4% of the total cells, were recovered.

### Statistical sequence analysis of the isolated clones

**Figure 2:**
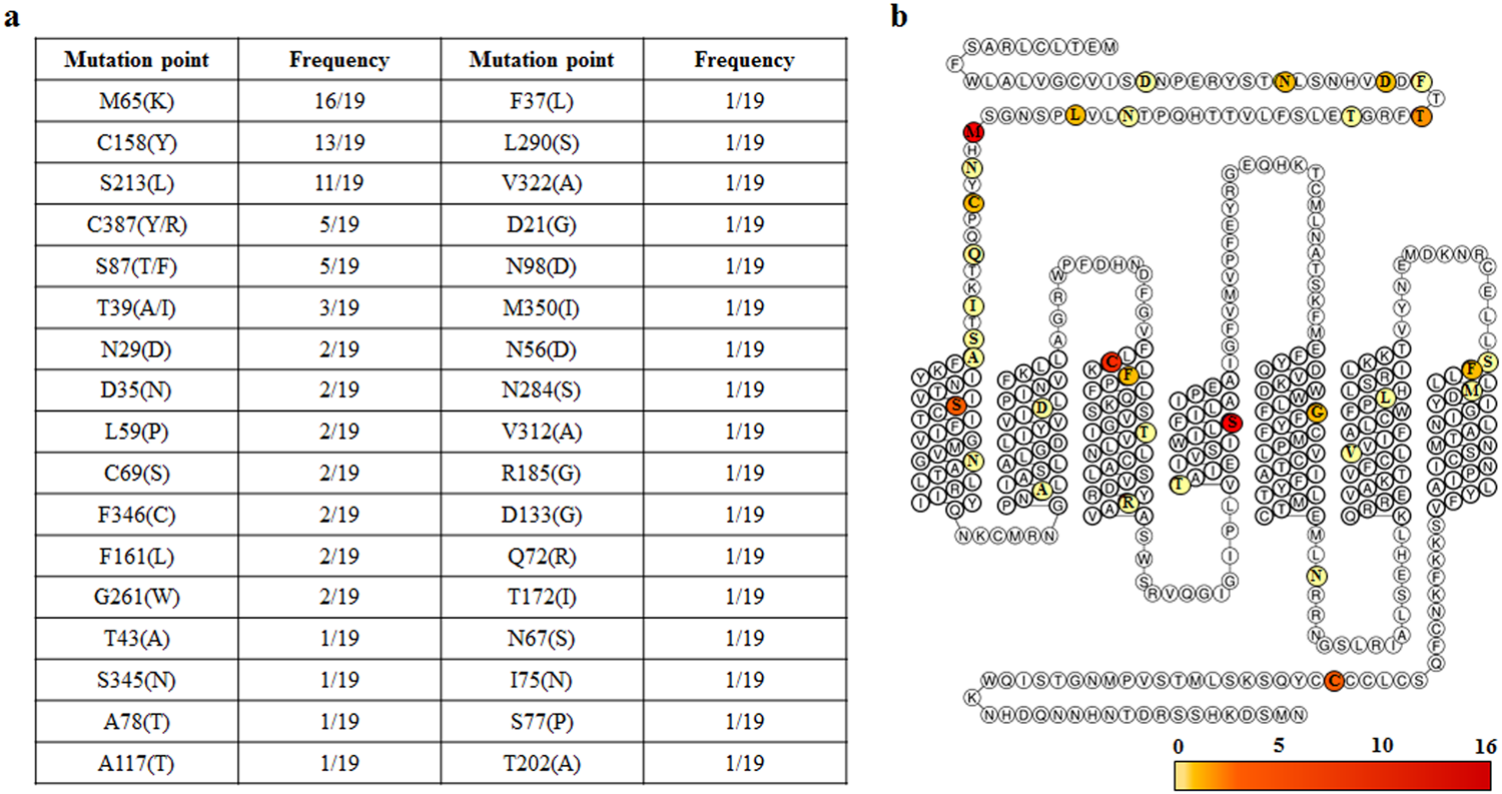
Statistical sequence analysis of the isolated clones and snake plot of the identified mutation points of ET_A_. (**a**) The mutations observed in the isolated clones are listed according to their frequencies. (**b**) Snake plot of the isolated mutation points of ET_A_. The colors of the circles indicate the frequency of mutation (Fig. 2a), as represented at the bottom with scale bar.

After TA cloning of the isolated clones using the pGEM-T vector, the sequences of 20 different plasmids selected during white colony screening were analyzed using T7 and SP6 promoter primers (Supplementary Table S1). The sequences of 19 clones were analyzed; clone number 20 was not sequenced due to failure of the sequence analysis (data not shown). Clone number 7 and 13 were confirmed to be the same clone. And the 34 mutation points were confirmed *via* statistical analysis of the sequencing results, and 13 mutation points were appeared at least twice (Fig. 2a). As shown in Fig. 2b, these mutation points, which may influence the binding of ET-1, are represented in the snake plot of ET_A_ (www.gpcrdb.org). More than half of these mutations were located at the extracellular N-terminus region and the entrance region of TM3 and TM7 (Fig. 2b). The remaining mutations were widely spread in the TM regions or in the intracellular domains.

### Investigation of ET-1 binding activities and structural integrities of the individual P9-ET_A_ mutants

**Figure 3:**
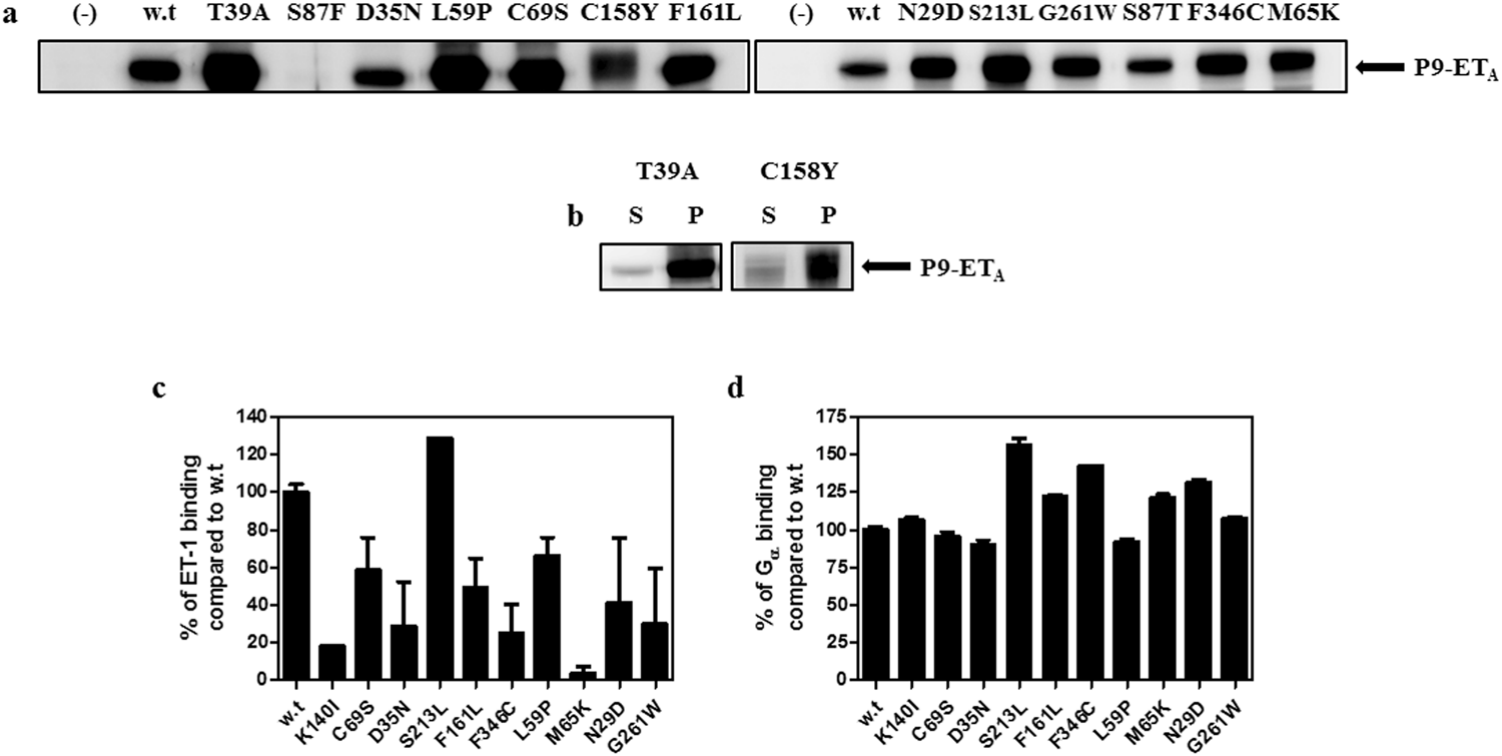
Expression of the individual mutants and the relative affinities of the mutants against ET-1 and G_αi3_. (**a**) Western blot analysis of the crude extracts from *E. coli* expressing P9-ET_A_ harboring each single amino acid substitution using anti-P9 antibodies. The lanes are described as follows. (−): crude extract prior to induction, w.t: crude extract of wild-type after induction, others: crude extracts of indicated mutants after induction. The full-length blots are represented in Supplementary Figure S8. (**b**) Western blot analysis of the supernatant (S) and pellet (P) fractions after centrifugation of cell lysates expressing T39A and C158Y mutants. The full-length blots are represented in Supplementary Figure S9. (**c**) ET-1 binding activities of the mutants compared to wild type P9-ET_A_. The purified mutants were analyzed for their ET-1 binding activities using 5 μM bET-1 and SA-HRP. “w.t” represents wild type P9-ET_A_, and the others represent the indicated mutants of P9-ET_A_. The error bar represents the standard error of the mean (SEM). (**d**) G_αi3_ binding activities of the mutants compared to the wild type. The interactions between the mutants and immobilized G_αi3_ were analyzed using 1 μM mutant and anti-P9 antibody. “w.t” represents wild type P9-ET_A_, and the others represent the indicated mutants of P9-ET_A_. The error bar represents the SEM.

To examine the effects of the identified mutations on their interactions with ET-1, ET_A_ mutants containing each identified mutation were prepared and their direct interactions with ET-1 were evaluated. We selected 12 mutation sites which appeared more than twice and were located at the extracellular or transmembrane regions for further study. The C387 mutation, which was observed five times in the mutants, was not included for further study because the mutation site was located in the intracellular region. The individual mutants composed of N29D, D35N, T39A, L59P, M65K, C69S, S87F, S87T, C158Y, F161L, S213L, G261W, and F346C were expressed in *E. coli* using a P9-fusion system, and their expression levels were determined (Fig. 3a). Among them, mutations that resulted in low expression (S87F; Fig. 3a) or inclusion body formation (T39A and C158Y; Fig. 3b) were not examined further. Wild type P9-ET_A_ and 10 different mutants, including K140I, which has been identified to be involved in ET-1 binding^20, 21^, were successfully expressed and purified (Supplementary Figs S1–S6).

We then reconstituted the proteins with APG, which was synthesized by the conjugation of alkyl and glucosyl groups to the carboxylic acid groups of poly-γ-glutamic acid and which facilitates the stabilization of P9-ET_A_ in its active conformation^27, 28^. Then, the ET-1 binding activities of the mutants compared to wild type P9-ET_A_ were investigated (Fig. 3c). To measure the relative binding activities of the purified proteins to ET-1, we applied 5 μM ET-1 to the immobilized proteins; this concentration is sufficient to cover the maximal binding capacity (B_max_) of the receptors because it is about 100-fold higher than the apparent equilibrium dissociation constant (K_D_) of the wild type P9-ET_A_ complex with APG against ET-1 (60 nM)^27^. As shown in Fig. 3c, all the mutants except S213L showed reduced binding to ET-1 compared to wild type P9-ET_A_. The control mutant containing the previously identified K140I mutation showed significantly reduced interaction with ET-1, suggesting that our system worked well as designed and that the identified mutation sites, except S213L, were involved in the interaction with ET-1. Notably, the M65K mutation, which was observed with the highest frequency in the sequence analysis (Fig. 2a), showed the lowest binding activity with ET-1.

It is possible that these mutations destabilize the native conformation of ET_A_ and that the reduced interaction with ET-1 results from conformational defects rather than direct involvement of the mutations in ET-1 binding. To examine the structural integrity of these mutant proteins, the interactions between the mutants and G_αi3_ were measured. The canonical signaling cascade triggered by ET-1 is mediated by G_αq_^29^. However, purified ET_A_ and ET_A_ expressed in mammalian cells can interact with various G_α_ proteins including G_αi3_^30, 31^. Therefore, we investigated whether the selected mutations affected the structural integrity of the protein by measuring the binding activities of the mutants to G_αi3_ compared to wild type P9-ET_A_. As shown in Fig. 3d, all the mutants showed similar or almost identical binding activities to the G_αi3_ protein compared to wild type P9-ET_A_, indicating that these mutations directly reduced the affinity to ET-1 rather than inducing structural distortions.

### Influences of the mutations on the intracellular response

**Figure 4:**
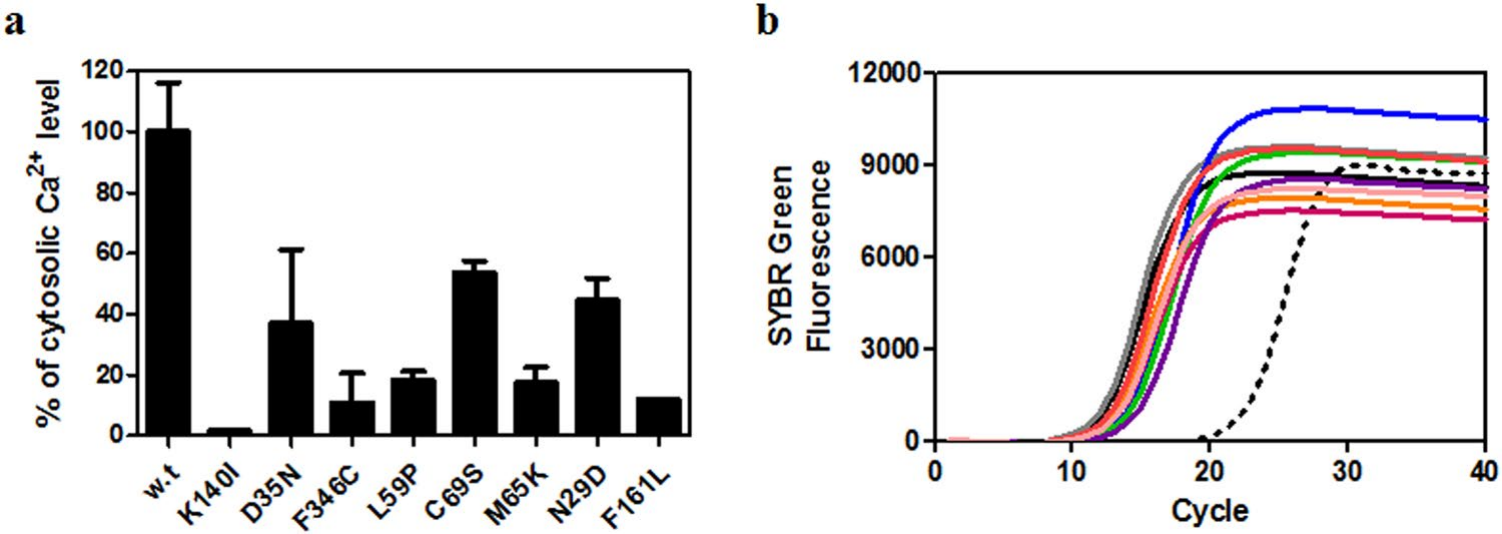
Measurement of cytosolic Ca^2+^ levels. (**a**) Measurement of cytosolic Ca^2+^ levels of CHO-K1 cells expressing wild type and mutant ET_A_ after treatment with ET-1. After transient expression of the mutants in the CHO-K1 cell line by transfection of the pCMVTag3B vector including each mutant as well as wild type ET_A_, the cells were incubated with 5 μM Fura-2-AM followed by incubation with 1 μM ET-1. The cytosolic Ca^2+^ level was estimated by measuring the ratio of the fluorescence intensities emitted at 510 nm at two distinct excitation wave-lengths, 340 nm and 380 nm. The percentage was calculated based on the ratio of wild type ET_A_ as 100% and the ratio of CHO-K1, which is not transfected, as 0%. “w.t” represents wild type ET_A_, and the others represent the indicated mutants of ET_A_. The error bar represents the SEM. (**b**) RT-PCR of cDNA from transfected CHO-K1 cells. After preparation of the total RNA from CHO-K1 cells harboring each mutant and wild type ET_A_, cDNA was obtained by reverse-transcription PCR using ET_A_ gene-specific primers. The cDNA was amplified by PCR reaction with SYBR green dye, and the SYBR green fluorescence signals of amplified DNA from non-transfected CHO-K1 (black dotted line), CHO-K1 harboring wild type ET_A_ (black solid line), K140I (gray line), N29D (blue line), D35N (green line), L59P (orange line), M65K (red line), C69S (magenta line), F161L (violet line), and F346C (pink line) were recorded in each PCR cycle.

The reduced affinity of ET_A_ to ET-1 for the identified mutations suggests that these mutations may retard ET-1-dependent intracellular signaling, such as the G_q_ signaling cascade. Signal transduction *via* G_q_, the major ET-1 stimulating signal cascade of ET_A_, accompanies the increment of cytosolic Ca^2+^ levels through Ca^2+^ release from the endoplasmic reticulum (ER) *via* the inositol-1,4,5 triphosphate (IP_3_) receptor^29^. To examine the effects of the mutations on the ET-1-dependent signaling cascade, we used a cytosolic Ca^2+^-sensitive fluorescent dye, Fura-2-AM, to measure the ET-1 dependent change of the intracellular Ca^2+^ levels of CHO cells that transiently expressed wild type or mutant ET_A_s. As shown in Fig. 4a, all the cells expressing the mutants showed notably decreased cytosolic Ca^2+^ concentrations compared to cells expressing wild type ET_A_ after stimulation by treatment with 1 μM ET-1. The effect of the K140I mutation appeared to be the most dramatic because the cytosolic Ca^2+^ level was almost identical to the level of naïve CHO cells after stimulation with ET-1 treatment. The cells expressing the F346C, L59P, M65K, and F161L mutants showed 80% to 90% decreased Ca^2+^ response compared to the cells expressing wild type ET_A_; the D35N, C69S, and N29D mutants exhibited 50% to 60% decreased Ca^2+^ response. The mRNA levels of mutated ET_A_ in the transformed CHO cells, measured by real-time PCR, were almost identical to the wild type ET_A_ expressing cells (Fig. 4b). Furthermore, the protein expression levels of ET_A_ variants in the transformed CHO cells were almost identical likewise the identical mRNA levels indicating that the mRNA levels are directly correlated with the expression levels of the proteins in the transformed CHO cells (Supplementary Fig. S7). These results indicated that the mutations that have reduced affinity to ET-1 also show retarded ET-1-dependent signaling.

## Discussion

In this study, we developed a biochemical strategy to identify the ET-1 binding sites of ET_A_ by a protein engineering method called “directed degeneration”. The ET_A_ variant library was successfully constructed using standard error-prone PCR (error rate: 0.46%). Through flow cytometric screening of the ET_A_ library on the basis of the fluorescence signals derived from GFP fusion at the C-terminus of ET_A_, and through binding of biotinylated ET-1 followed by streptavidin-PE labeling, GFP-positive and PE-negative clones possessing no internal stop codon and low binding activity to ET-1 were isolated. DNA sequencing of the selected clones revealed 34 mutation points that potentially affected ET-1 recognition. Among them, 13 mutation points were observed repeatedly, suggesting significant roles of these mutations in ET-1 binding. Particularly, the M65K mutation appeared in 16 out of 19 clones. Individual ET_A_ mutants with each identified mutation, except for T39, S87, C158, and C387, were successfully expressed and purified along with the K140I mutant.

Nine of ten mutant ET_A_s (N29D, D35N, L59P, M65K, C69S, K140I, F161L, G261W, and F346C) showed considerably reduced ET-1 binding activities compared to wild type ET_A_. Furthermore, the mutants likely have no significant structural distortions because they had very similar or slightly higher G_αi3_ binding activities compared to wild type ET_A_. To investigate the quantitative rate constants (k_a_ and k_d_) and the K_D_ of ET-1 for the ET_A_ variants compared with wild type ET_A_, surface plasmon resonance (SPR) experiments were carried out using XPR36 (Bio-Rad, USA) equipment. In the SPR experiments, P9-ET_A_ was immobilized very poorly on sensor chips functionalized with nitrilotriacetic acid (NTA) or carboxymethyl (CM) groups (data not shown). We believe that these immobilization problems may derive from steric hindrance of the poly-histidine tag at the C-terminus and the low accessibility of carboxymethyl groups to the exposed lysine residues.

One mutation (G261W) that showed reduced affinity to ET-1 substituted glycine for tryptophan in the center of the fifth transmembrane helix (TM5), as depicted in Fig. 2b. This substitution may directly affect the interaction with ET-1. Alternatively, the Trp substitution may affect the structure of ET_A_ by disturbing the compactness of the transmembrane helices by decreasing the degree of structural freedom and increasing the occupied space, thereby reducing the affinity of ET_A_ against ET-1. Likewise, the C69S mutation was assumed to destabilize ET_A_ because this substitution prevents the formation of a disulfide bridge with C341 at extracellular loop 3 (ECL3)^24^. We propose that the C69S and G261W mutants destabilized the structure of ET_A_, resulting in low binding affinity to ET-1. Identification of the C69S and G261W mutants by this strategy provides further evidence that the “directed degeneration” method is effective to identify mutations that affect ligand binding as well as mutations that destabilize protein structures.

**Figure 5:**
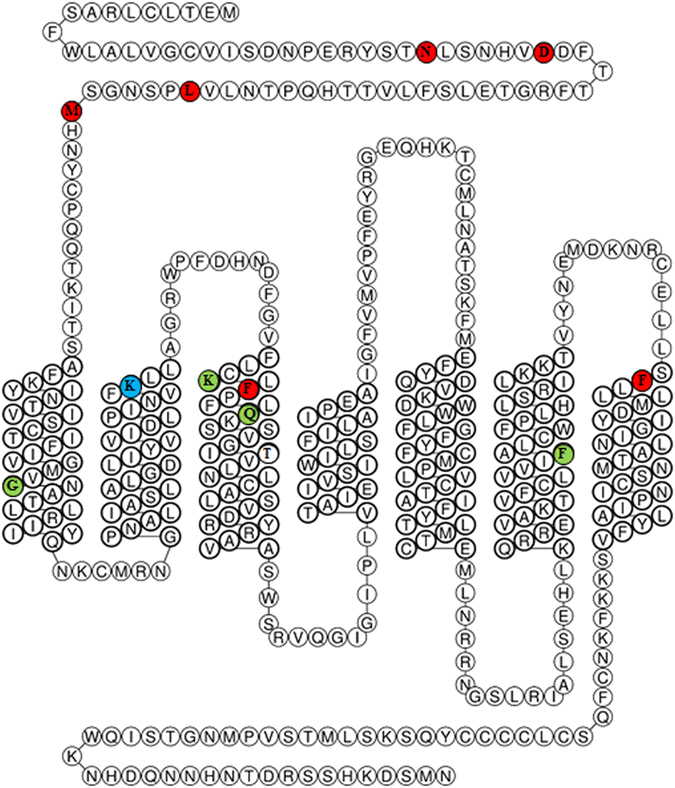
Snake plot of ET-1 binding sites. The ET-1 binding sites identified by this study (red circles) and previous reports (green circles) are represented in a snake plot of ET_A_. The K140 position, which was identified in previous reports and was used in this study as a control, is represented by a blue circle.

It is confirmed that the six novel mutations (N29D, D35N, L59P, M65K, F161L, and F346C) decreased the binding of ET-1 and also inhibited downstream signaling of ET_A_ stimulated by ET-1 in mammalian cells. The newly identified ligand binding sites of ET_A_ in this study and the amino acids identified by previous reports are represented in Fig. 5 ^20, 21^. It should be noted that several amino acids in the extracellular N-terminus domain of ET_A_ were first identified as crucial residues for ET-1 binding. Additionally, F161 and F346, which are critical for the interaction with ET-1, are located at the entrance regions of TM3 and TM7, respectively, similarly to K140 (entrance of TM2). Considering the high solubility of the ET-1 peptide (>1 mg/mL), our results indicate that the binding site of ET-1 is mainly located in the extracellular domain of ET_A_. Recently, a crystal structure of endothelin receptor type B (ET_B_), which lack the N-terminus region (1–87 amino acids), complex with ET-1 has been reported^32^. In this structure, the extracellular region of ET_B_ is widely involved in ET-1 binding, which is in good agreement with our results. The three C-terminus residues of ET-1 (I19, I20, and W21) bind to the hydrophobic pocket of ET_B_ consisted of I157 (TM2), P178 (TM3), V185 (TM3), F240 (ECL2), L277 (TM5), W336 (TM6), L339 (TM6), and Y369 (TM7). The F161 (TM3) and F346 (TM7) of ET_A_ which are identified as ET-1 binding sites of ET_A_ are correspond to the P178/V185 and Y369 of ET_B_, respectively, and they might form hydrophobic ET-1 binding pocket likewise ET_B_. Although the structure of ET_B_ with ET-1 could not provide a precise role of the N-terminus domain in ET-1 binding, the amino acids in the N-terminus domain of ET_B_, which are correspond to the amino acids (N29, D35, L59, and M65) of ET_A_, which are identified as ET-1 binding sites in this study, may have an important role to recognize ET-1.

The “directed degeneration” method is distinct from previous biochemical approaches^20, 21^, which focus on localized regions for mutagenesis study; this is because our method introduces random mutations in any part of the protein, and mutants with decreased binding affinity to the ligand are selected. Therefore, the strategy of this study provided a novel recognition mode of ET_A_ against its ligand. Structural investigation has provided much valuable information about protein including ligand interaction modes; however, the exact structural information of ET_A_ bound with ET-1 has not been reported to date due to the lack of high resolution structures and ambiguity of molecular model structures^22–24^. The newly identified residues of ET_A_ that are implicated in the interaction with ET-1 will contribute to the construction of a novel molecular ET-1 binding model that very closely approximates the native structure of ET_A_ bound with ET-1. Conclusively, the identified ET-1 binding mode of ET_A_ will facilitate understanding of the signal transduction cascades of ET_A_ as well as the design of novel drugs targeting ET_A_. Furthermore, the “directed degeneration” method provides a convenient molecular tool to dissect the interaction modes of ligands with their receptors.

## Methods

### Materials

*Dpn*I, *Hin*dIII, *Bam*HI, Phusion DNA polymerase, T5 exonuclease, *Taq* DNA ligase, dATP, dGTP, dCTP, dTTP, and T4 DNA ligase were obtained from New England Biolabs (UK). *Taq* DNA polymerase was purchased from Takara (Japan) and Hot Start *Taq* DNA polymerase was obtained from Bioneer (Korea). Synthesis of oligonucleotides and DNA sequencing were performed by Bioneer and Cosmogenetech (Korea). Polyclonal P9 antibody was prepared from mouse anti-serum using P9 protein as an antigen (Ab Frontier, Korea). C-terminally biotinylated endothelin-1 (bET-1) was synthesized by both of Peptron and LugenSci (Korea). Endothelin-1 (ET-1) was obtained from Sigma (USA). pGEM-T vector was purchased from Promega (USA). Phycoerythrin-conjugated streptavidin (SA-PE) and HRP-conjugated streptavidin (SA-HRP) were obtained from BD Biosciences (USA) and Thermo Scientific (USA), respectively. Trypsin-EDTA, F-12 media, FBS, Lipofectamine 2000, Fura-2-AM, and antibiotic-antimycotic solution were obtained from ThermoFisher (USA). Cell culture dishes were purchased from SPL (Korea). Ni-NTA resin was obtained from Qiagen (Germany). All other consumable reagents were purchased from Sigma (USA).

### ET_A_ library construction

Error-prone PCR of ET_A_ was performed using a primer set (ET_A_ Fw: 5′-tggtgccgcgcggctcccggggagaaaccctttgcctcagggcatcc-3′ and ET_A_ Rv: 5′-gaaaagttcttctcct ttactcatgttcatgctgtccttatggctgct-3′) with a target 0.5% error rate (1 error/200 bp) according to a previous report^33^. After PCR amplification of GFP with a primer set (GFP Fw: 5′-agcagccataaggacagcatgaacatgagtaaaggagaagaacttttc-3′ and GFP Rv: 5′-ttaatggtgatggtgatggtga gaagcttccttggatagttcatccatgccatg-3′), the PCR products of the randomized ET_A_ and GFP were assembled by PCR using the ET_A_ Fw and GFP Rv primers. After digestion of pP9 vector with *Hin*dIII for linearization, the PCR product of randomized ET_A_-GFP was inserted into the linearized pP9 by the Gibson cloning method according to the following process^34^. 1.33X master mix composed of PEG-8000, MgCl_2_, TrisCl pH7.5, DTT, NAD^+^, dNTPs, T5 exonuclease, *Taq* DNA ligase, and Phusion DNA polymerase was incubated with the PCR product and the linearized vector for 4 h at 50 °C, followed by transformation of the reaction mixture into *E. coli* BL21(DE3). The diversity of the library was estimated by titrating the number of colonies on LB agar supplemented with 100 μg/mL of ampicillin in a square plate. The mutation rate of the constructed library was analyzed by DNA sequencing.

### Flow cytometric screening

*E. coli* BL21(DE3) cells harboring plasmids for ET_A_ variants were inoculated into 100 mL of LB media containing 100 μg/mL ampicillin. The cells were grown at 37 °C until the OD_600nm_ reached 0.5 to 0.6, followed by induction at 25 °C for 4 h with 0.5 mM IPTG. After harvesting the cells by centrifugation at 5,000 × g for 20 min, spheroplasts were obtained by osmotic shock and lysozyme treatment as previously described^35^. The resulting spheroplasts were incubated with 0.5 μM bET-1 at room temperature for 1 h, washed with PBS, and subsequently incubated with 10 nM SA-PE under the same incubation conditions. After washing, the fluorescently labeled spheroplasts were analyzed by flow cytometry (S3 cell sorter, Bio-Rad, USA) and cell populations displaying high GFP signals and low PE signals were sorted.

### TA cloning of the isolated clones

The sorted spheroplasts were re-cloned with the pGEM-T vector for sequence analysis. ET_A_ genes in the isolated clones were amplified by PCR using a primer set (5′-tgccgcgcggctcccgggcggaaaccctttgcctcagggc-3′ and 5′-ttaatggtgatggtgatggtgagaa gcttcgttcatgctgtccttatggctgc-3′) and Hot Start *Taq* polymerase. After the PCR products and pGEM-T Easy Vector were ligated by incubating at 4 °C over-night with T4 DNA ligase, the reaction mixture was transformed into *E. coli* (DH5α strain) and spread on a LB agar plate containing 100 μg/mL ampicillin, 0.5 mM IPTG, and 80 μg/mL X-gal.

### Statistical sequence analysis of the isolated clones

After TA cloning of the isolated clones, the sequences of 20 different plasmids selected during white colony screening were analyzed using T7 and SP6 promoter primers. Based on the sequences of 19 clones (clone number 20 was not sequenced due to failure of the sequence analysis (data not shown)), the appeared each single mutation was statistically arranged by the frequency.

### Site-directed mutagenesis

Site-directed mutagenesis was performed to generate individual P9-ET_A_ mutants by PCR using wild-type pP9-ET_A_ as a template and the primer set described in Supplementary Table S2, followed by incubation with *Dpn*I. It was confirmed by sequence analysis that the C158Y, S87F, D35N, and N29D mutants were successfully generated; other mutants, however, were not successfully generated. To address this problem, we designed new primer sets using conventional mutagenesis method involving two-step PCR containing K140I, a common mutant identified in a previous study^20, 21^, to use as a control mutant as described in Supplementary Table S3. The first and second fragments were amplified by PCR using the T7 promoter primer/Frag1 Rv primer set and the Frag2 Fw/T7 terminator primer set, respectively. The two DNA fragments were assembled by PCR using the T7 promoter and T7 terminator primer. The PCR products were inserted into the linearized pP9 vector by *Hin*dIII digestion using the Gibson cloning method. The sequences of the mutants were analyzed using the T7 promoter and T7 terminator primers.

### Preparation of P9-ET_A_ wild-type and mutants

Expression and purification of the P9-ET_A_ wild type and mutants were performed as described previously^25^. Briefly, expression of the wild type and mutants in *E. coli* BL21(DE3) strain was induced by the addition of 0.5 mM IPTG when the optical density of the culture at 600 nm reached 0.5 to 0.6. After induction, the cells were incubated at 25 °C for 4 h and harvested by centrifugation. The cell pellets from 1.0 L of culture were resuspended in 20 mL of buffer A (25 mM Tris–HCl, pH 7.8) containing 1 mM PMSF, and the cells were lysed using an M-110P microfludizer (Microfluidics, USA). Cell debris was removed from the lysate by centrifugation at 12,000 × g for 20 min before the membrane fraction was recovered by ultracentrifugation at 100,000 × g for 1 h. The pellet containing the membrane proteins was homogenized with buffer B (0.5% sarkosyl in buffer A) at 4 °C for 3 h, and the insoluble materials were removed by centrifugation at 30,000 × g for 30 min. The supernatant containing solubilized membrane proteins was loaded onto a Ni-NTA agarose column that was pre-equilibrated with buffer B. After washing the column with 20 mM imidazole in buffer B, the bound proteins were eluted with 300 mM imidazole in buffer B. The buffer of the eluted protein fraction was exchanged with buffer B using a PD-10 desalting column (GE, USA) to remove excess imidazole. The prepared proteins were stabilized in their active conformation with APG as previously described^27, 28^. The proteins were stored with 10% glycerol at −80 °C until further use.

### ELISA for ET-1 binding activity of the mutants

5 μg of each of the P9-ET_A_ wild-type and mutants were immobilized on a 96-well medium binding plate (Corning, USA) by incubating at room temperature for 2 h. After extensive washing, each well was blocked with 5% (w/v) skim milk followed by incubation with 5 μM bET-1 at room temperature for 2 h. After extensive washing, the amount of bET-1 bound with the ET_A_ variant was estimated by color development using SA-HRP and TMB; then 2.5 N H_2_SO_4_ was added to quench the reaction. The absorption at 450 nm of each well was measured using a multi-plate reader (Triad multimode detector, DYNEX Technologies, USA).

### ELISA for G_αi3_ binding activity of the mutants

After G_αi3_ protein was prepared as previously described^36^, 1 μg of G_αi3_ was immobilized on each well of a 96-well medium binding plate by incubating at room temperature for 2 h. After blocking the vacant space of each well with 5% skim milk, 1 μM of each mutant and the P9-ET_A_ wild type were treated and incubated at room temperature for 2 h, followed by extensive washing. Anti-P9 and HRP-conjugated anti-mouse antibody were sequentially incubated at room temperature for 1 h. After extensive washing, the amount of P9-ET_A_ was measured by color development of TMB. The reaction was quenched by adding 2.5 N H_2_SO_4_ and the absorption at 450 nm of each well was measured using the multi-plate reader.

### CHO cell culture and cytosolic Ca^2+^ level measurement

We tested whether the mutations could affect cytosolic Ca^2+^ levels by ET-1 compared to the wild type using Fura-2-AM, which is a cytosolic Ca^2+^-sensitive fluorescent dye. The procedure to measure the variations in cytosolic Ca^2+^ levels was adapted from a previous method^37^. The genes of wild type and mutant ET_A_ in the pP9 vector were subcloned into the pCMVTag3B mammalian expression vector. The genes were amplified by PCR using a primer set (5′-gaggatctgagcccgggcggatccgattataaaga tgatgatgataaaatggaaaccctttgcctcagggc-3′ and 5′-aggtaccgggccccccctcgagtcattagttcatgctgtccttat ggc-3′). The PCR products were ligated into the pCMVTaq3B vector using *Bam*HI and *Hind*III restriction endonuclease sites by the Gibson cloning method. CHO-K1 cells from the American Type Culture Collection (USA) were cultured at 37 °C in a 5% CO_2_ humidified incubator, and maintained in Ham’s F-12 nutrient mix, GlutaMAX media supplemented with 10% (v/v) FBS, and 1x antibiotic-antimycotic solution in a 6-well cell culture plate until the cells reached 80% confluence. 2.5 μg of plasmids containing the mutant or wild type ET_A_ was transfected into the cells in each well using Lipofectamine 2000. After harvesting trypsin-digested cells by centrifugation at 200 × g for 3 min, the cells were resuspended in Hepes-buffered medium (HBM) consisting of 20 mM Hepes pH7.4, 103 mM NaCl, 4.8 mM KCl, 1.2 mM KH_2_PO_4_, 1.2 mM MgSO_4_, 0.5 mM CaCl_2_, 25 mM NaHCO_3_, and 15 mM glucose; the cells were then incubated at room temperature with 5 μM Fura-2-AM for 40 min. After addition of 1 μM ET-1 to the 10^5^ transfected cells, the emissions at 510 nm for two excitation wavelengths (340 nm and 380 nm) were recorded for 100 sec using a fluorescence spectrophotometer (FS-2, Scinco, USA). The cytosolic Ca^2+^ level was evaluated by obtaining the ratio of the average fluorescence intensity excited at 340 nm to the intensity excited at 380 nm.

### Real-time PCR

After preparation of the total RNA of CHO-K1 cells harboring each ET_A_ mutant or wild type ET_A_, the complementary DNA (cDNA) was obtained by reverse transcription-PCR using an ET_A_ gene-specific primer set (5′-gcagaagtcctcggtg-3′ and 5′-ccaatc gcttcaggaatg-3′) and 110 ng of total RNA. The cDNA was amplified by PCR with SYBR green dye, and the signals of SYBR green incorporated into double-stranded DNA were monitored by a CFX96 Real-Time PCR detection system (Bio-Rad, USA).

## Electronic supplementary material

Supplementary information
