# Supplementary material for: Determination of the endothelin-1 recognition sites of endothelin receptor type A by the directed-degeneration method
Source: Sci Rep. 2017 Aug 8;7:7577. doi: 10.1038/s41598-017-08096-6 (PMC5548930; doi:10.1038/s41598-017-08096-6)
Supplement: Supplementary file 1 — Supplementary information [file 41598_2017_8096_MOESM1_ESM.pdf]

Determination of the endothelin-1 recognition sites of endothelin receptor type A by the directed-degeneration method

Seong-Gu Han[1], Sanghwan Ko[1], Won-Kyu Lee[1,2], Sang Taek Jung[1*] & Yeon Gyu Yu[1*]

[1]Department of Chemistry, Kookmin University, 861-1 Jeongneung-dong, Seongbuk-gu, Seoul, 136-702, Republic of Korea

[2]New Drug Development Center, Osong Medical Innovation Foundation, Osong Sengmyung-Ro 123, Osong-eup, Heungdeok-gu, Cheongju-si, Chungbuk, Republic of Korea

[*]To whom correspondence should be addressed

Department of Chemistry, Kookmin University, 861-1 Jeongneung-dong, Seongbuk-gu, Seoul, 136-702, Korea

Yeon Gyu Yu

E-mail: ygyu@kookmin.ac.kr

Sang Taek Jung

E-mail: sjung@kookmin.ac.kr

Clone No.	Nucleic acid No.	Mutation	Amino acid change	Clone No.	Nucleic acid No.	Mutation	Amino acid change
1	127-129	ACA→GCA	T43A	11	193-195	ATG→AAG	M65K
	193-195	ATG→AAG	M65K		259-261	TCT→TTT	S87F
	472-474	TGC→TAC	C158Y		472-474	TGC→TAC	C158Y
	637-639	TCG→TTG	S213L		637-639	TCG→TTG	S213L
	1033-1035	AGT→AAT	S345N		850-852	AAC→AGC	N284S
	1117-1119	AGC→AGT	Silent		934-936	GTT→GCT	V312A
					1159-1161	TGC→CGC	C387R
2	193-195	ATG→AAG	M65K	12	115-117	ACT→GCT	T39A
	232-234	GCT→ACT	A78T		193-195	ATG→AAG	M65K
	250-252	ACT→ACC	Silent		259-261	TCT→ACT	S87T
	349-351	GCG→ACG	A117T		505-507	GTG→GTA	Silent
	505-507	GTG→GTA	Silent		553-555	AGA→GGA	R185G
	619-621	GTC→GTT	Silent		619-621	GTC→GTT	Silent
	1159-1161	TGC→TAC	C387Y		1036-1038	TTC→TGC	F346C
3	109-111	TTC→CTC	F37L	13	175-177	CTA→CCA	L59P
	193-195	ATG→AAG	M65K		193-195	ATG→AAG	M65K
	472-474	TGC→TAC	C158Y		472-474	TGC→TAC	C158Y
	637-639	TCG→TTG	Silent		637-639	TCG→TTG	S213L
	868-870	TTG→TCG	L290S		1159-1161	TGC→TAC	C387Y
	1207-1209	ACA→ACG	Silent				
4	193-195	ATG→AAG	M65K	14	115-117	ACT→GCT	T39A
	472-474	TGC→TAC	C158Y		193-195	ATG→AAG	M65K
	637-639	TCG→TTG	Silent		259-261	TCT→ACT	S87T
	658-660	GAA→GAG	Silent		505-507	GTG→GTA	Silent
	964-966	GTT→GCT	V322A		553-555	AGA→CGA	Silent
					619-621	GTC→GTT	Silent
					1036-1038	TTC→TGC	F346C
5	193-195	ATG→AAG	M65K	15	163-165	ACT→ACC	Silent
	472-474	TGC→TAC	C158Y		202-204	TAT→TAC	Silent
	637-639	TCG→TTG	S213L		205-207	TGC→AGC	C69S
	1159-1161	TGC→TAC	C387Y		259-261	TCT→ACT	S87T
					397-399	GAT→GGT	D133G
					481-483	TTC→CTC	F161L
					946-948	GCT→GCC	Silent
6	85-87	AAT→GAT	N29D	16	193-195	ATG→AAG	M65K
	103-105	GAT→AAT	D35N		472-474	TGC→TAC	C158Y

	193-195<br>244-246<br>472-474<br>637-639<br>997-999<br>1117-1119	ATG→AAG<br>ATT→ATC<br>TGC→TAC<br>TCG→TTG<br>TAT→TAC<br>AGC→AGT	M65K<br>Silent<br>C158Y<br>S213L<br>Silent<br>Silent		637-639	TCG→TTG	S213L
**7**	175-177<br>193-195<br>472-474<br>637-639<br>1159-1161	CTA→CCA<br>ATG→AAG<br>TGC→TAC<br>TCG→TTG<br>TGC→TAC	L59P<br>M65K<br>C158Y<br>S213L<br>C387Y	**17**	193-195<br>214-216<br>472-474<br>514-516<br>637-639<br>781-783	ATG→AAG<br>CAG→CGG<br>TGC→TAC<br>ACC→ATC<br>TCG→TTG<br>GGG→TGG	M65K<br>Q72R<br>C158Y<br>T172I<br>S213L<br>G261W
**8**	61-63<br>193-195<br>292-294<br>472-474<br>637-639	GAT→GGT<br>ATG→AAG<br>AAT→GAT<br>TGC→TAC<br>TCG→TTG	D21G<br>M65K<br>N98D<br>C158Y<br>S213L	**18**	193-195<br>472-474<br>637-639<br>781-783<br>817-819<br>1114-1116	ATG→AAG<br>TGC→TAC<br>TCG→TTG<br>GGG→TGG<br>ATC→ATT<br>GTG→GTT	M65K<br>C158Y<br>S213L<br>G261W<br>Silent<br>Silent
**9**	163-165<br>205-207<br>259-261<br>505-507<br>619-621<br>1048-1050	ACT→ACC<br>TGC→AGC<br>TCT→ACT<br>GTG→GTA<br>GTC→GTT<br>ATG→ATA	Silent<br>C69S<br>S87T<br>Silent<br>Silent<br>M350I	**19**	85-87<br>103-105<br>115-117<br>199-201<br>223-225<br>229-231<br>481-483<br>511-513<br>604-606	AAT→GAT<br>GAT→AAT<br>ACT→ATT<br>AAC→AGC<br>ATT→AAT<br>TCA→CCA<br>TTC→CTC<br>ATC→ATT<br>ACT→GCT	N29D<br>D35N<br>T39I<br>N67S<br>I75N<br>S77P<br>F161L<br>Silent<br>T202A
**10**	166-168<br>193-195<br>472-474<br>637-639	AAT→GAT<br>ATG→AAG<br>TGC→TAC<br>TCG→TTG	N56D<br>M65K<br>C158Y<br>S213L	**20**	-	-	-

Supplementary Table S1. Sequence analysis results of the isolated clones by FACS screening after TA cloning.

**ETA_M65K_Fw (41mer)**	**ETA_M65K_Rv (41mer)**
**5’-CCTACCCAGCAATGGCTCAAAGCACAACTATTGCCCACAGC-3’**	**5’-GCTGTGGGCAATAGTTGTGCTTTGAGCCATTGCTGGGTAGG-3’**
**ETA_C158Y_Fw (40mer)**	**ETA_C158Y_Rv (40mer)**
**5’-GACTTTGGCGTATTTCTTTACAAGCTGTTCCCCTTTTTGC-3’**	**5’-GCAAAAAGGGGAACAGCTTGTAAAGAAATACGCCAAAGTC-3’**
**ETA_S213L_Fw (40mer)**	**ETA_S213L_Rv (40mer)**
**5’-GTCTCCATCTGGATCCTGTTGTTTATCCTGGCCATTCCTG-3’**	**5’-CAGGAATGGCCAGGATAAACAACAGGATCCAGATGGAGAC-3’**
**ETA_S87T_Fw (43mer)**	**ETA_S87T_Rv (43mer)**
**5’-CAAATACATTAACACTGTGATAACTTGTACTATTTTCATCGTG-3’**	**5’-CACGATGAAAATAGTACAAGTTATCACAGTGTTAATGTATTTG-3’**
**ETA_S87F_Fw (43mer)**	**ETA_S87F_Rv (43mer)**
**5’-CAAATACATTAACACTGTGATATTTTGTACTATTTTCATCGTG-3’**	**5’-CACGATGAAAATAGTACAAAATATCACAGTGTTAATGTATTTG-3’**
**ETA_T39A_Fw (43mer)**	**ETA_T39A_Rv (43mer)**
**5’-CAATCATGTGGATGATTTCACCGCTTTTCGTGGCACAGAGCTC-3’**	**5’-GAGCTCTGTGCCACGAAAAGCGGTGAAATCATCCACATGATTG-3’**
**ETA_N29D_Fw (37mer)**	**ETA_N29D_Rv (37mer)**
**5’-CCTGAGAGATACAGCACAGATCTAAGCAATCATGTGG-3’**	**5’-CCACATGATTGCTTAGATCTGTGCTGTATCTCTCAGG-3’**
**ETA_D35N_Fw (40mer)**	**ETA_D35N_Rv (40mer)**
**5’-CAAATCTAAGCAATCATGTGAATGATTTCACCACTTTTCG-3’**	**5’-CGAAAAGTGGTGAAATCATTCACATGATTGCTTAGATTTG-3’**
**ETA_L59P_Fw (40mer)**	**ETA_L59P_Rv (40mer)**
**5’-CAACCCACTAATTTGGTCCCACCCAGCAATGGCTCAATGC-3’**	**5’-GCATTGAGCCATTGCTGGGTGGGACCAAATTAGTGGGTTG-3’**
**ETA_C69S_Fw (45mer)**	**ETA_C69S_Rv (45mer)**
**5’-CAATGGCTCAATGCACAACTATAGCCCACAGCAGACTAAAATTAC-3’**	**5’-GTAATTTTAGTCTGCTGTGGGCTATAGTTGTGCATTGAGCCATTG-3’**
**ETA_F346C_Fw (40mer)**	**ETA_F346C_Rv (40mer)**
**5’-CCGATGTGAATTACTTAGTTGCTTACTGCTCATGGATTAC-3’**	**5’-GTAATCCATGAGCAGTAAGCAACTAAGTAATTCACATCGG-3’**
**ETA_F161L_Fw (41mer)**	**ETA_F161L_Rv (41mer)**
**5’-**	**5’-**

GCGTATTTCTTTGCAAGCTGCTCCCCTTTTTGCAGAAGTCC-3’	GGACTTCTGCAAAAAGGGGAGCAGCTTGCAAAGAAATACGC-3’
**ETA_G261W_Fw (42mer)**	**ETA_G261W_Rv (42mer)**
5’-GTAAAGGACTGGTGGCTCTTCTGGTTCTATTTCTGTATGCCC-3’	5’-GGGCATACAGAAATAGAACCAGAAGAGCCACCAGTCCTTTAC-3’

Supplementary Table S2. Firstly designed primer sets for site directed mutagenesis.

**ETA_T39A_Frag2 Fw (24mer)**	**ETA_T39A_Frag1 Rv (24mer)**
**5'-GATGATTTCACCGCTTTTCGTGGC-3'**	**5'-GCCACGAAAAGCGGTGAAATCATC-3'**
**ETA_F161L_Frag2 Fw (24mer)**	**ETA_F161L_Frag1 Rv (24mer)**
**5'-CTTTGCAAGCTGCTCCCCTTTTTG-3'**	**5'-CAAAAAGGGGAGCAGCTTGCAAAG-3'**
**ETA_C69S_Frag2 Fw (22mer)**	**ETA_C69S_Frag1 Rv (22mer)**
**5'-GCACAACTATAGCCCACAGCAG-3'**	**5'-CTGCTGTGGGCTATAGTTGTGC-3'**
**ETA_L59P_Frag2 Fw (24mer)**	**ETA_L59P_Frag1 Rv (24mer)**
**5'-CTAATTTGGTCCCACCCAGCAATG-3'**	**5'-CATTGCTGGGTGGGACCAAATTAG-3'**
**ETA_S87T_Frag2 Fw (25mer)**	**ETA_S87T_Frag1 Rv (25mer)**
**5'-CACTGTGATAACTTGTACTATTTTC-3'**	**5'-GAAAATAGTACAAGTTATCACAGTG-3'**
**ETA_S213L_Frag2 Fw (23mer)**	**ETA_S213L_Frag1 Rv (23mer)**
**5'-CTGGATCCTGTTGTTTATCCTGG-3'**	**5'-CCAGGATAAACAACAGGATCCAG-3'**
**ETA_F346C_Frag2 Fw (28mer)**	**ETA_F346C_Frag1 Rv (28mer)**
**5'-GAATTACTTAGTTGCTTACTGCTCATGG-3'**	**5'-CCATGAGCAGTAAGCAACTAAGTAATTC-3'**
**ETA_M65K_Frag2 Fw (25mer)**	**ETA_M65K_Frag1 Rv (25mer)**
**5'-CAATGGCTCAAAGCACAACTATTGC-3'**	**5'-GCAATAGTTGTGCTTTGAGCCATTG-3'**
**ETA_G261W_Frag2 Fw (23mer)**	**ETA_G261W_Frag1 Rv (23mer)**
**5'-GGTGGCTCTTCTGGTTCTATTTC-3'**	**5'-GAAATAGAACCAGAAGAGCCACC-3'**
**ETA_K140I_Frag2 Fw (24mer)**	**ETA_K140I_Frag1 Rv (24mer)**
**5'-CAATGTATTTATTCTGCTGGCTGG-3'**	**5'-CCAGCCAGCAGAATAAATACATTG-3'**

Supplementary Table S3. Secondly designed primer sets for site directed mutagenesis.

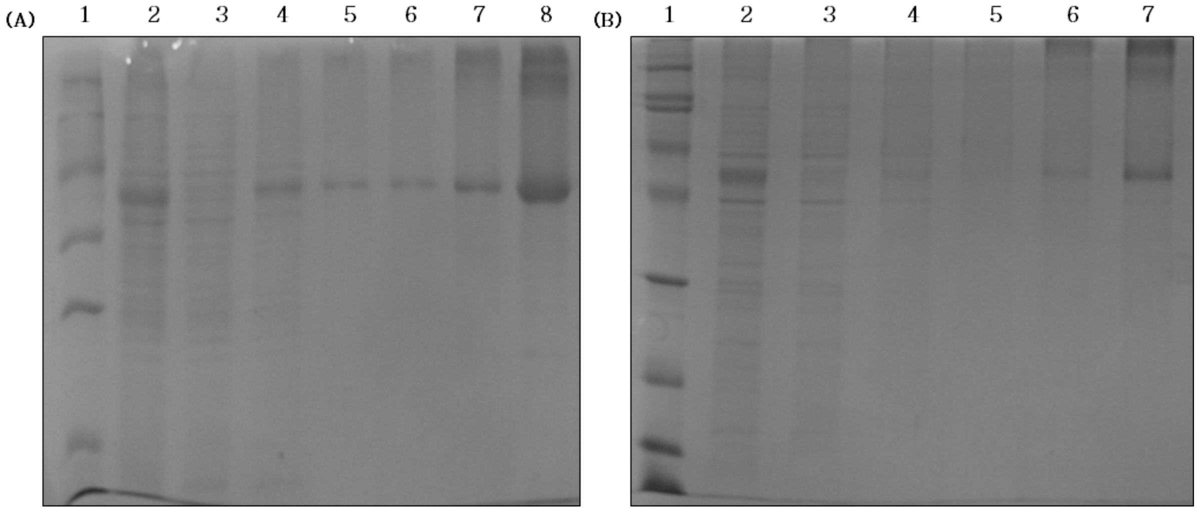

Supplementary Figure S1. Preparation of the P9-ET$_A$-K140I (A) and M65K (B) mutants. The lanes are described as the below. 1 : Molecular size marker, 2 : Membrane fraction, 3 : Flow through fraction of Ni-NTA chromatography, 4 : Washing fraction with 20 mM imidazole contained buffer, 5 : 1st elution fraction with 300 mM imidazole, 6 : 2nd elution fraction with 300 mM imidazole, 7 : 3rd elution fraction with 300 mM imidazole, 8 : 4th elution fraction with 300 mM imidazole.

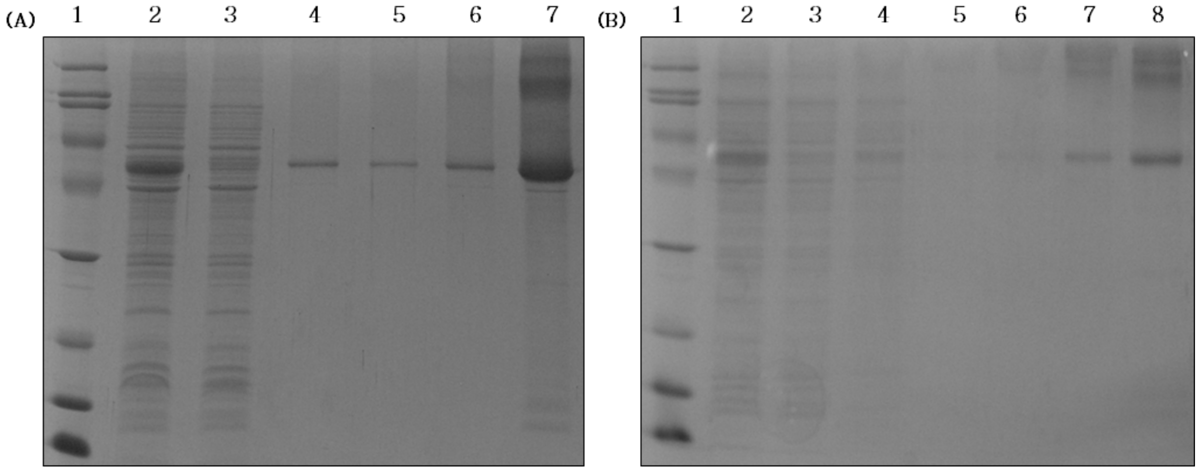

Supplementary Figure S2. Preparation of the P9-ET$_A$-S213L (A) and L59P (B) mutants. The lanes are described as the below. 1 : Molecular size marker, 2 : Membrane fraction, 3 : Flow through fraction of Ni-NTA chromatography, 4 : Washing fraction with 20 mM imidazole contained buffer, 5 : 1st elution fraction with 300 mM imidazole, 6 : 2nd elution fraction with 300 mM imidazole, 7 : 3rd elution fraction with 300 mM imidazole, 8 : 4th elution fraction with 300 mM imidazole.

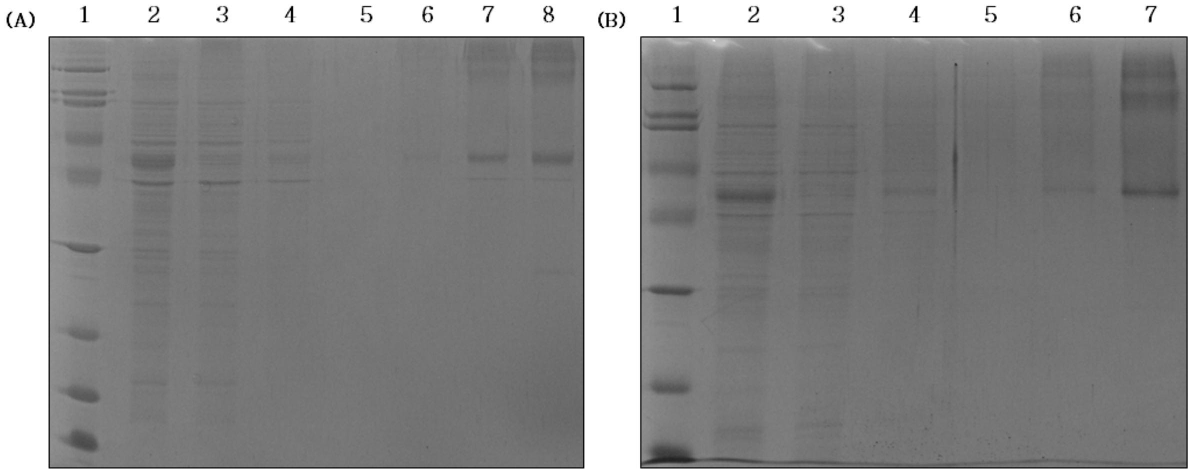

Supplementary Figure S3. Preparation of the P9-ET$_A$-G261W (A) and F346C (B) mutants. The lanes are described as the below. 1 : Molecular size marker, 2 : Membrane fraction, 3 : Flow through fraction of Ni-NTA chromatography, 4 : Washing fraction with 20 mM imidazole contained buffer, 5 : 1st elution fraction with 300 mM imidazole, 6 : 2nd elution fraction with 300 mM imidazole, 7 : 3rd elution fraction with 300 mM imidazole, 8 : 4th elution fraction with 300 mM imidazole.

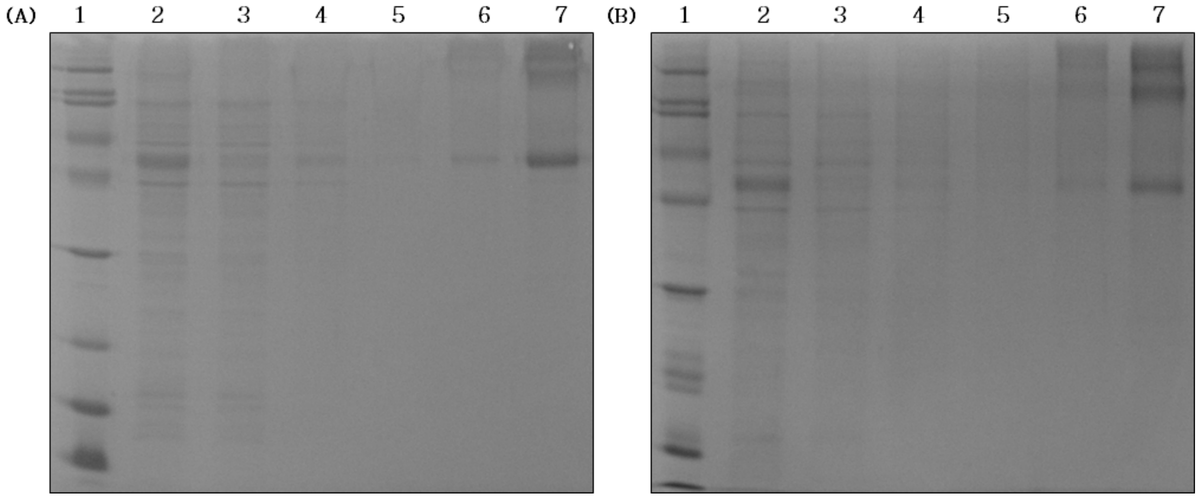

Supplementary Figure S4. Preparation of the P9-ET_A-F161L (A) and C69S (B) mutants. The lanes are described as the below. 1 : Molecular size marker, 2 : Membrane fraction, 3 : Flow through fraction of Ni-NTA chromatography, 4 : Washing fraction with 20 mM imidazole contained buffer, 5 : 1st elution fraction with 300 mM imidazole, 6 : 2nd elution fraction with 300 mM imidazole, 7 : 3rd elution fraction with 300 mM imidazole, 8 : 4th elution fraction with 300 mM imidazole.

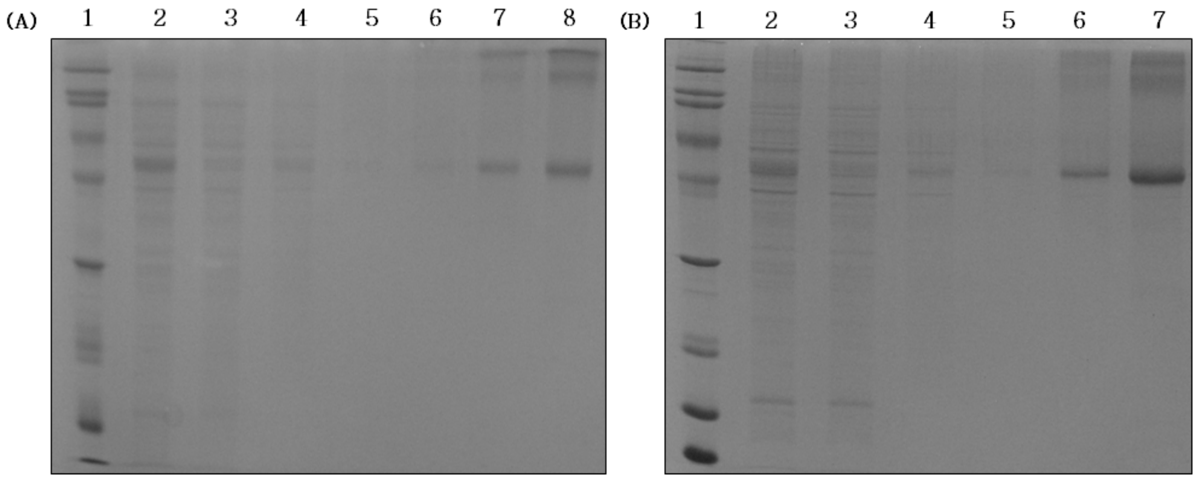

Supplementary Figure S5. Preparation of the P9-ET$_A$-N29D (A) and D35N (B) mutants. The lanes are described as the below. 1 : Molecular size marker, 2 : Membrane fraction, 3 : Flow through fraction of Ni-NTA chromatography, 4 : Washing fraction with 20 mM imidazole contained buffer, 5 : 1st elution fraction with 300 mM imidazole, 6 : 2nd elution fraction with 300 mM imidazole, 7 : 3rd elution fraction with 300 mM imidazole, 8 : 4th elution fraction with 300 mM imidazole.

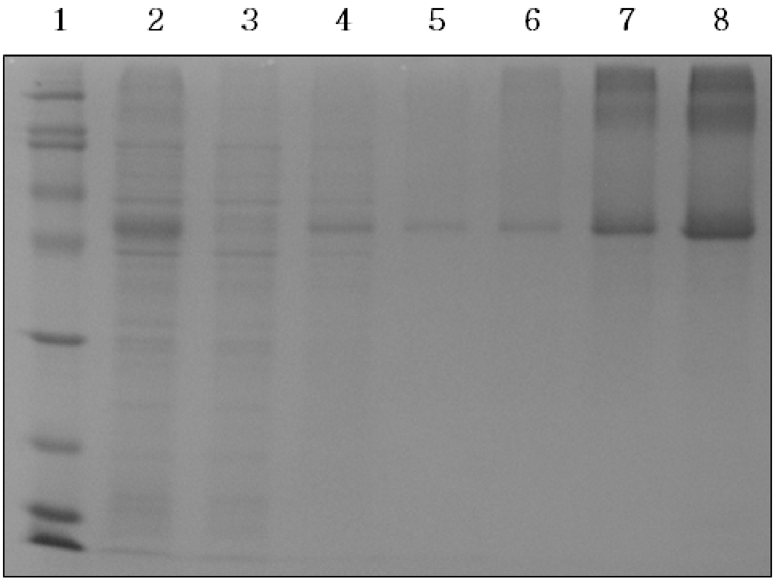

Supplementary Figure S6. Preparation of the wild-type P9-ET$_A$. The lanes are described as the below. 1 : Molecular size marker, 2 : Membrane fraction, 3 : Flow through fraction of Ni-NTA chromatography, 4 : Washing fraction with 20 mM imidazole contained buffer, 5 : 1st elution fraction with 300 mM imidazole, 6 : 2nd elution fraction with 300 mM imidazole, 7 : 3rd elution fraction with 300 mM imidazole, 8 : 4th elution fraction with 300 mM imidazole.

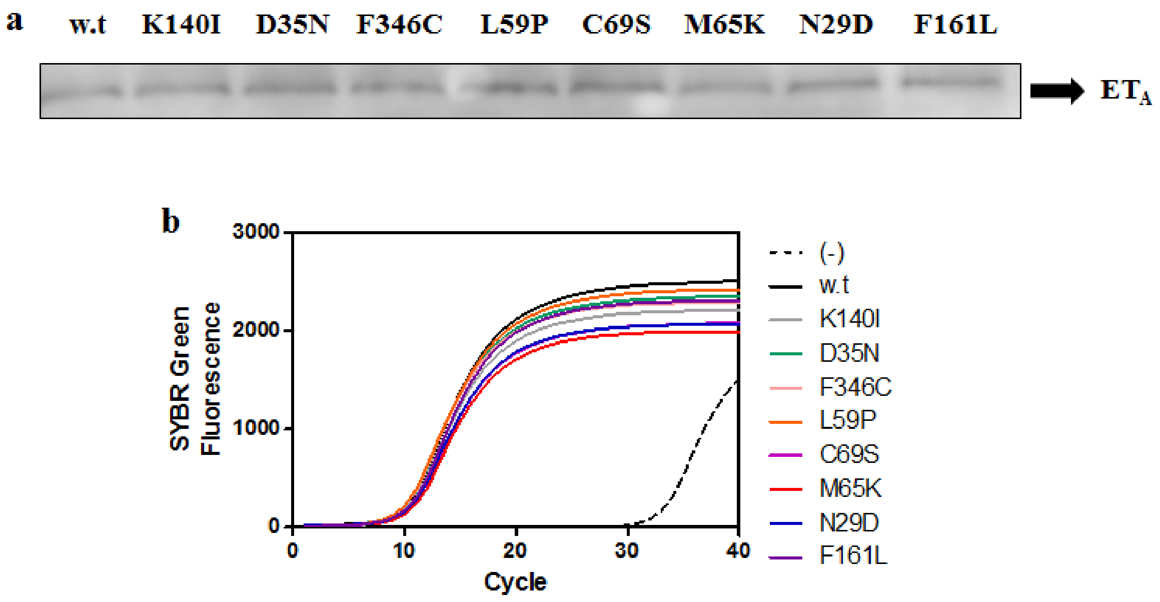

Supplementary Figure S7. Correlation between the expression level of ET$_A$ protein and mRNA. (a) Western blot analysis of CHO cells harboring each ET$_A$ mutant or wild type ET$_A$ using anti-ET$_A$ antibodies (Abcam, UK). The lanes are described as follows. w.t: CHO cell expressing wild type ET$_A$, others: CHO cells expressing indicated mutants. (b) RT-PCR of cDNA from transfected CHO-K1 cells. After preparation of the total RNA from CHO-K1 cells harboring each mutant and wild type ET$_A$, cDNA was obtained by reverse-transcription PCR using ET$_A$ gene-specific primers. The cDNA was amplified by PCR reaction with SYBR green dye, and the SYBR green fluorescence signals of amplified DNA from non-transfected CHO-K1 (black dotted line), CHO-K1 harboring wild type ET$_A$ (black solid line), K140I (gray line), N29D (blue line), D35N (green line), L59P (orange line), M65K (red line), C69S (magenta line), F161L (violet line), and F346C (pink line) were recorded in each PCR cycle.

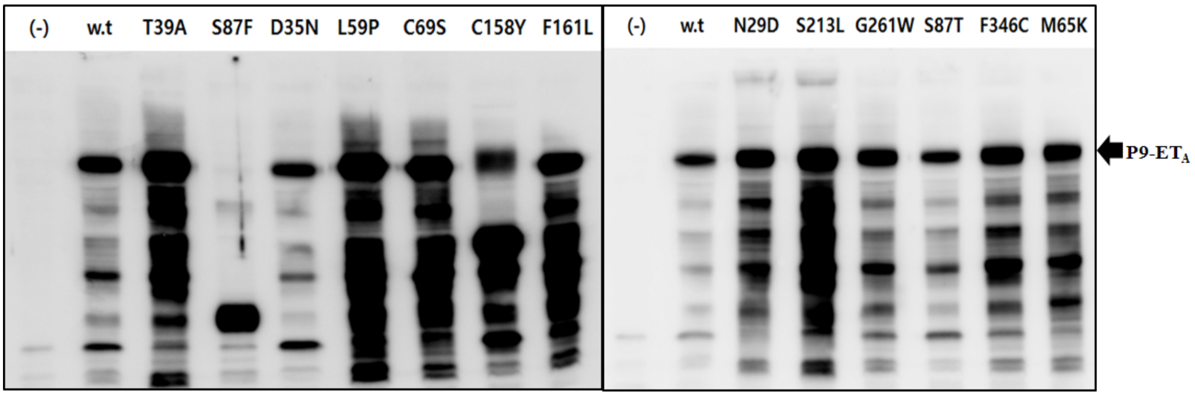

Supplementary Figure S8. Full-length blots for analysis of expression of the individual mutants. Western blot analysis of the crude extracts from *E. coli* expressing P9-ET_A harboring each single amino acid substitution using anti-P9 antibodies. The lanes are described as follows. (-): crude extract prior to induction, w.t: crude extract of wild-type after induction, others: crude extracts of indicated mutants after induction.

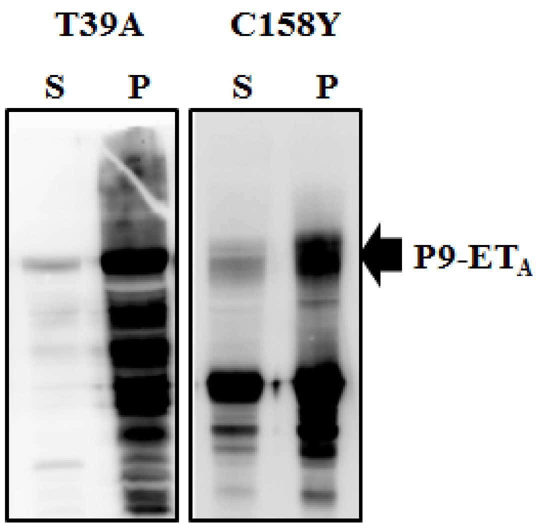

Supplementary Figure S9. Full-length blots for analysis of inclusion body expression of T39A and C158Y mutants. Western blot analysis of the supernatant (S) and pellet (P) fractions after centrifugation of cell lysates expressing T39A and C158Y mutants using anti-P9 antibodies.
